# Efficient Sky Dehazing by Atmospheric Light Fusion

**DOI:** 10.3390/s20174893

**Published:** 2020-08-29

**Authors:** Jaouad Hajjami, Thibault Napoléon, Ayman Alfalou

**Affiliations:** 1 Forssea Robotics, 130 rue de Lourmel, 75015 Paris, France; jaouad@forssea-robotics.fr; 2 L@bISEN Yncréa Ouest, 20 rue Cuirassé Bretagne, 29200 Brest, France; thibault.napoleon@isen-ouest.yncrea.fr

**Keywords:** image processing, single image dehazing, atmospheric light fusion

## Abstract

In this article, we present a new method of dehazing based on the Koschmieder model, which aims to restore an image that has been affected by haze. The difficulty is to improve the estimation of the transmission and the atmospheric light that generally suffer from the nonhomogeneity and the random variability of the environment. The keypoint is to enhance the dehazing of very bright regions of the image in order to improve the treatment of the sky that is often overestimated or underestimated compared to the rest of the scene. The approach proposed in this paper is based on two main contributions: 1. an L0 gradient optimization function weighted by a set of Gaussian filters and based on an iterative algorithm for optimization convergence. Unlike the existing methods using a single value of the atmospheric light for the whole image, our method uses a set of values neighboring an initial estimated value. The fusion is then applied based on Laplacian and Gaussian pyramids to combine all the relevant information from the set of images constructed from atmospheric lights and improves the contrast to recover the colors of the sky without any artifacts. Finally, the results are validated by three criteria: an autocorrelation score (*ZNCC*), a similarity measure (*SSIM*) and a visual criterion. The experiments carried out on two datasets show that our approach allows a better dehazing of the images with higher *SSIM* and *ZNCC* measurements but also with better visual quality.

## 1. Introduction

Image restoration is one of the fundamental issues in image processing taken under degraded conditions, such as fog or turbidity in underwater environments. Several solutions have been proposed in the literature such as regularization of histogram [1,2], CLAHE [3], etc. In order to implement dehazing on real applications like visual positioning for offshore companies, that suffer from haze and fog, we are particularly interested in dehazing techniques with the following two constraints: 1. using only a single input image with no additional data. 2. computation time less than ten seconds.

Dehazing an image usually consists of using the diffusion model of Koschmieder [4]. Among the first methods to have dealt with this problem, He’s [5] method is based on the dark channel (DCP) which is then refined with the Laplacian matrix to obtain the transmission. This solution is robust but very expensive in calculation time and it is based on a statistical observation that claims that in a small window of the image there is at least one dark channel, which is not always true and does not lead to a good estimation of the transmission. Several other methods derived from the DCP. For example, He [6] proposed an improvement of the transmission with a smoothing guided filter to preserve the contours. Others have used a median filter [7], a median of a median filter [8] or bilateral filter [9]. The weak points of these methods are the calculation time, which is quite high and generates several errors in areas where the image is very bright or close to the color of the haze. This is due not only to their approach but to the diffusion model [4] itself. On the other hand, Meng [10] tried to use another paradigm based on the optimization of the transmission by an iterative algorithm, but their method generates color distortions in the dark areas of the image. The approach of Ancuti [11] is based on the weighted fusion of two images derived from the haze image: contrast enhancement and color correction. However, their algorithm works mostly on images with degraded illumination.

In order to improve the performance of these methods, we propose to fuse several values of the atmospheric light to better estimate the haze, or the transmission, and obtain results closer to reality as the atmospheric light appears twice in the diffusion formula. We also improve the radiance by a gradient optimization function weighted by a Gaussian filter of the transmission. We summarize in three points our contributions:

- efficient estimation of $A_{s}$ (scalar atmospheric light) used as a central value for the fusion,
- optimization of two regularization terms of the transmission,
- radiance J(x) computed by two possible methods:
  – total fusion of all possible values of $A_{s}=\left[0,255\right]$ weighted by Gaussian coefficients,
  – limited weightless fusion of the neighborhood values of $A_{s}$ estimated in the first step.

## 2. Contributions

### 2.1. Estimation of Atmospheric Light $A_{s}$

In Koschmieder’s diffusion model, the atmospheric light $A_{s}$ is by definition the color of the haze. Several works have attempted to estimate it, in general, either by selecting the pixel manually or by taking the clearest region of the image [12]. To improve this estimation, He [5] took the darkest pixel of the 0.1% brightest pixels. Narasimhan [13] used two images to retrieve the orientation of $A_{s}$ as the atmospheric light vector corresponds to the difference between the two images. Shwartz [14] goes in the same direction by exploiting two or more images with different polarization states to estimate the variation of fog and then deduce the value of $A_{s}$ from that. On the other hand, the approach of Fattal [15] is based on the intersection between the RGB channels of several windows designated by the user. The common weak point between these methods is the estimation of $A_{s}$ in the “burnt” areas causing by several sources such as artificial light, sun, sky, etc. To overcome these problems, we propose in this section a new and more robust approach to these situations.

The atmospheric light $A_{s}$ plays an important role in the formation of the final image, or the radiance, and especially on its contrast. For this we propose two key improvements for an efficient and fast estimation of this scalar: first, the estimation of $A_{s}$ is done in the grayscale version of the input image $I_{gray}\left(x\right)$, second, in its downsampled version. This can be expressed by the following formula:

(1) $$A_{s}=max\left\{h{\left(x\right)}_{win}⊛\left[R_{scale}\circ I_{gray}\left(x\right)\right]\right\},$$

where $R_{scale}$ corresponds to the resized image operator by a factor scale<1 and $h{\left(x\right)}_{win}⊛$ is the application of the minimum filter h(x), which is an order-statistic filter [16], on a window of size win of an image I(x), which is in our case the resized image: $R_{scale}\circ I_{gray}\left(x\right)$.

The formulation of the Equation (1) is based on two facts:

1. the fog is best represented in the gray level image because the hazed images contain few colors and their contrast is very low. So, we propose to estimate it on this single channel for a homogeneous rendering especially in areas where the haze is more present.
2. “burned” areas of the image have a maximum pixel value (e.g., 255), which is often incorrect to be considered as the haze color. For this reason, the grayscale channel is then resized by reducing its size to further smooth burned areas (high light) by merging several pixels together.

In reality, a simple observation of the image will show that the haze occupies a large part of the scene, and resizing the image taken from this scene will bring an overall smoothing effect of the haze. Therefore, the value of $A_{s}$ will be considered as an average of all possible values.

**Figure 1:**
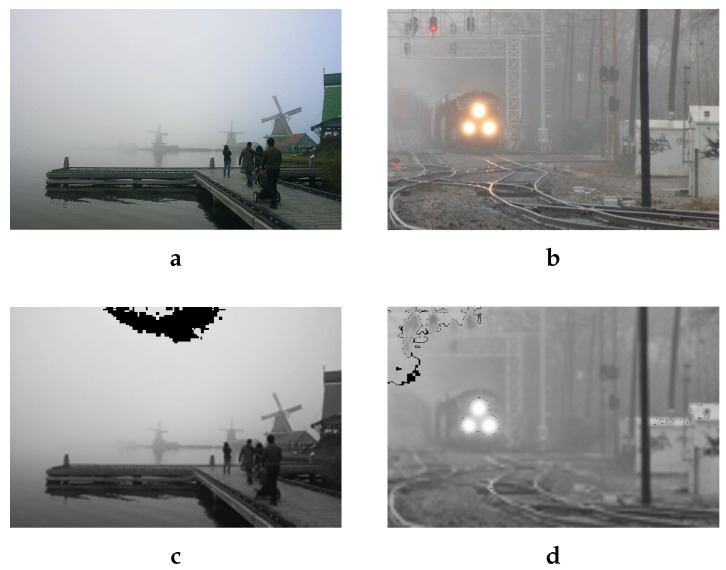
This Figure shows two examples of hazed images (first row) and black pixels showing the location of the estimated $A_{s}$ (second row). With scale=0.2 and win=10. (**a**): Hazy image-1. (**b**): Hazy image-2. (**c**): The location of the color of the haze in black pixels for the image (**a**). (**d**): The location of the color of the haze in black pixels for the image (**b**).

The true color of the haze $A_{s}$ corresponds to the pixel where the haze is densest. The Figure 1 highlights the areas of haze estimated with the Equation (1). The results show clearly that the proposed estimation of $A_{s}$ succeeds in locating the pixels with greatest depth corresponding to a denser haze. Indeed, for an infinite depth, we can show that the pixels of an image are equal to the atmospheric light $A_{s}$. By starting from the Koschmieder [4] model:

(2) $$I\left(x\right)=t\left(x\right)\left(J\left(x\right)-A_{s}\right)+A_{s}$$

where I(x) is the input image (the hazed image), J(x) is the output image (the dehazed image), t(x) is the transmission and it is correlated to the scene depth d(x): $t\left(x\right)=e^{-\gamma d(x)}$. If we have an infinite depth: d(x)→+∞, then the pixels of the image converge to the atmospheric light $A_{s}$:

(3) $$\lim_{d(x)\to +\infty }t\left(x\right)=\lim_{d(x)\to +\infty }e^{-\gamma d(x)}=0\Rightarrow \lim_{d(x)\to +\infty }I\left(x\right)=A_{s}$$

where γ>0 is the molar extinction coefficient.

This way of estimating $A_{s}$ is very fast, the Table 1 shows that our approach is faster than Meng [10] that uses the minimum filter h(x) on a full RGB image or by taking the darkest pixel of the 0.1% brightest pixels such as in [5,17].

Despite these improvements that can be made during the computation of $A_{s}$, it is not sufficient. In fact, the haze in all the areas of the image (with artificial light, clouds, sky, etc.) is estimated with the same constant $A_{s}$. We will see in the Section 3 how to give more importance to this variable through pyramidal fusion.

### 2.2. Estimation of the Transmission t(x)

**Figure 2:**
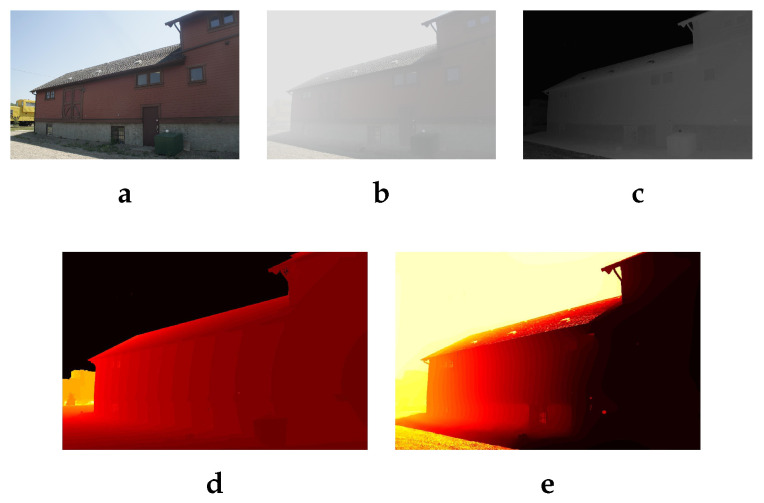
Optimization of the transmission: (**a**): haze-free image. (**b**): hazy image. (**c**): $t_{b}\left(x\right)$ the initialization of the transmission t(x). (**d**): ground truth depth [19]. (**e**): t(x) the transmission. As the haze is very often nonhomogeneous, the depth information is not sufficient for dehazing. For illustration purpose, we show both the ground truth depth (**d**) and the estimated transmission (**e**). The estimated transmission should be proportional to the depth map but also reflects the density of the haze. In the transmission (**e**), the right side is less hazy and very close to the camera, so the transmission is approaching zero (dark color) and on the left side; however, the haze is denser, so the transmission approaches one (white color). The sky area in the ground truth image (**d**) is missing the depth information; it is usually considered as undefined or as infinity. This is useless in dehazing, particularly, sky dehazing which is the main contribution of this paper. Therefore, for those two reasons (nonhomogeneity and invalid depth in sky regions) the depth information cannot be introduced in Equation (3) as it is defined in Koschmieder [4] model and it needs to be considered as a haze density for real image dehazing.

Transmission t(x) is based on the molar extinction γ and the depth of the scene. These parameters are very often not given and cannot be easily estimated from a single camera image. Therefore, we define the transmission as the “depth” of the haze and not as the depth of the scene. For this, we propose to introduce a new optimization function that is defined with a weighted smoothing operator. First of all, we use the boundary constraint from Meng [10] who considers fog as nonhomogeneous and unknown, defined as follows:

(4) $$0\leq t_{b}\left(x\right)\leq t\left(x\right)\leq 1,$$

with $t_{b}\left(x\right)$ is the minimum value of t(x) computed as: $t_{b}\left(x\right)=min\left\{max\left(\frac{A_{s}-I\left(x\right)}{A_{s}-C_{0}},\frac{A_{s}-I\left(x\right)}{A_{s}-C_{1}}\right),1\right\}$ where $C_{0}=20$ and $C_{1}=300$ are the radiance limits. As stated by Meng [10], in Figure 2, this comes from the fact that the scene radiance J(x) is always bounded by $C_{0}$ and $C_{1}$: $C_{0}<J\left(x\right)<C_{1}$.

The Figure 2 shows an example of $t_{b}\left(x\right)$ image that will be used to initialize our transmission estimation process to obtain a map that looks like a fog’s depth map. Our estimation process is being characterized by:

- keeping only the main contours defined by the greatest depth difference between two regions of the image. Given that no depth information is available, our estimation is based on the difference of intensity between two homogeneous regions.
- using a Gaussian filter for smoothing the areas where the variation of the depth (e.g., intensity variation) is small.

To integrate these two criteria, we introduce them as a prior representing two regularization terms. The first term corresponds to the L0 norm of the contours of $t_{b}\left(x\right)$ and the second term corresponds to the Gaussian smoothing of $t_{b}\left(x\right)$. After that we propose a model based on those two prior pieces of information about the unknown t(x). This model is then optimized using an alternating minimization algorithm with a total variation (TV) regularization as it is presented by Wang [18].

### 2.3. Formulation

We assume that the transmission t(x) is defined in a bounded variation in [0,1] over the domain $\Omega \in R^{2}$. The goal is to minimize the following energy:

(5) $$\min_{t}{∥t-t_{b}∥}_{2}^{2}$$

where ${∥t-t_{b}∥}_{2}^{2}$ is the TV term to keep the function from oscillating in accordance with other parameters. The final solution converges towards a unique solution close to the observed image, $t_{b}\left(x\right)$ in our case.

In order to converge the Equation (5) to a unique solution, we have to stabilize it with two regularization terms:

(6) $$\min_{t}\lambda {∥t-t_{b}∥}_{2}^{2}+{\left|\nabla t\right|}_{0}+{∥H⊛t∥}_{1}$$

with:

1. λ is the regularization parameter.
2. ${\left|\nabla t\right|}_{0}=|\delta_{x}t|+|\delta_{y}t|$: the “norm” zero corresponds to the number of times the magnitude $|\delta_{x}t|+|\delta_{y}t|$ is not black.
3. ${∥H⊛t∥}_{1}$: the application of the Gaussian filter *H* on the transmission *t* for smoothing areas with a low gradient.

In order to have a stable and convergent minimization of this kind of objective function, Wang [18] proposed an alternate solution by introducing auxiliary variables into the cost function. For our Equation (6), it would be two variables *W* and $w=(w_{h},w_{v})$, where *W*, $w_{h}$ and $w_{v}$ are the approximations of the transmission, the horizontal gradient and the vertical gradient, respectively.

By integrating those variables, the new cost function becomes:

(7) $$\min_{t,W,w}\lambda {∥t-t_{b}∥}_{2}^{2}+\frac{\beta }{2}\left({∥\delta_{x}t-w_{h}∥}_{2}^{2}+{∥\delta_{y}t-w_{v}∥}_{2}^{2}\right)+{\left|\nabla w\right|}_{0}+{∥H⊛W∥}_{1}+\frac{\beta }{2}{∥W-t∥}_{2}^{2}$$

The quadratic terms are used to keep the auxiliary variables close to their corresponding variables and they are measured in 0-norm and 1-norm to handle noise (other than white noise). β is a penalty parameter. Theoretically speaking, if β→∞, the solution of the cost Equation (7) converges to the cost Equation (6).

Solving the cost Equation (7) is done by simultaneously optimizing three subproblems with respect to *W*, *w* and *t*. Meaning that, we solve for each optimal *W*, *w* or *t* while considering the two others as constants.

**Solving *W***:

The first subproblem is the minimization over *W* by fixing *t* and *w* and it is given by:

(8) $$\min_{W}{∥H⊛W∥}_{1}+\frac{\beta }{2}{∥W-t∥}_{2}^{2}$$

Considering the approximation H⊛W≈W as we apply a Gaussian operator with smaller blurs, the problem becomes easier to solve. Thus it can be written as:

$$\min_{x}\left|x\right|+\frac{\beta }{2}{\left(x-cst\right)}^{2}$$

where cst is constant at this stage. This has a unique minimizer $x^{*}$:

$$x^{*}=max(|cst|-\frac{1}{\beta },0){\;}^{.}\;sign\left(cst\right)$$

where sign() is the sign function.

**Solving the intermediate transmission**$t_{i}$:

We introduce in this subproblem the intermediate transmission $t_{i}\left(x\right)$ in which we consider only the gradients *w*. We then solve the following problem:

(9) $$\min_{t_{i}}\lambda {∥t_{i}-t_{b}∥}_{2}^{2}+\frac{\beta }{2}\left({∥\delta_{x}t_{i}-w_{h}∥}_{2}^{2}+{∥\delta_{y}t_{i}-w_{v}∥}_{2}^{2}\right)+{\left|\nabla w\right|}_{0}$$

**Solving *w***: The second subproblem is the minimization over *w* by fixing $t_{i}$ and it is given by:

(10) $$\min_{w}{∥\delta_{x}t_{i}-w_{h}∥}_{2}^{2}+{∥\delta_{y}t_{i}-w_{v}∥}_{2}^{2}+\frac{2}{\beta }{\left|\nabla w\right|}_{0}$$

where ${\left|\nabla w\right|}_{0}$ corresponds to the number of times *w* is nonzero. This energy can be spatially decomposed where $w_{h}$ and $w_{v}$ could be estimated independently. Thus the above problem reaches its minimum under the following condition:

(11) $$w=\left(w_{h},w_{v}\right)=\left\{\begin{array}{cc} \left(0,0\right) & if\;\;{\left(\delta_{x}t_{i}\right)}^{2}+{\left(\delta_{y}t_{i}\right)}^{2}⩽\frac{2}{\beta }\\ \left(\delta_{x}t_{i},\delta_{y}t_{i}\right) & Otherwise \end{array}\right.$$

**Solving $t_{i}$**: The third subproblem is the minimization over $t_{i}$ by fixing *w*:

(12) $$\min_{t_{i}}\lambda {∥t_{i}-t_{b}∥}_{2}^{2}+\frac{\beta }{2}\left({∥\delta_{x}t_{i}-w_{h}∥}_{2}^{2}+{∥\delta_{y}t_{i}-w_{v}∥}_{2}^{2}\right)$$

We note that the objective Equation (12) is quadratic in $t_{i}$ and the optimal $t_{i}^{*}$ could be given by the normal equation:

(13) $$\left(\frac{2\lambda }{\beta }+\nabla w^{T}\nabla w\right)t_{i}=\frac{2\lambda }{\beta }t_{b}+\nabla w^{T}t_{i}$$

We can compute an optimal solution for $t_{i}\left(x\right)$ when it is resolved in the frequency domain, for speedup, by applying a two-dimensional discrete Fourier transform to the above equation. We then have the optimal solution $t_{i}^{*}$ as:

(14) $$t_{i}^{*}=F^{-1}\left\{\frac{\frac{2\lambda }{\beta }F\left(t_{b}\right)+F^{*}\left(\delta_{x}\right)F\left(w_{h}\right)+F^{*}\left(\delta_{y}\right)F\left(w_{v}\right)}{F^{*}\left(\delta_{x}\right)F\left(\delta_{x}\right)+F^{*}\left(\delta_{y}\right)F\left(\delta_{y}\right)}\right\}$$

where F is the Fast Fourier Transform (FFT) operator and $F^{*}$ is the complex conjugate.

**Solving the final transmission** t(x)

After solving the intermediate $t_{i}\left(x\right)$, we tackle here the final transmission t(x) by optimizing the following problem that includes t(x) in both of its terms from the original cost Equation (7) while considering the intermediate transmission $t_{i}\left(x\right)$ instead of $t_{b}\left(x\right)$:

(15) $$\min_{t}\lambda {∥t-t_{i}∥}_{2}^{2}+\frac{\beta }{2}{∥W-t∥}_{2}^{2}$$

Again, the above subproblem has the same form as the previous one, as in the cost Equation (12). Therefore, we apply the Fourier transform to its optimal solution which is the normal equation:

(16) $$\left(\frac{2\lambda }{\beta }+W^{T}W\right)t=\frac{2\lambda }{\beta }t_{i}+W^{T}t$$

After that we get the optimal transmission computed in the frequency domain as follows:

(17) $$t^{*}=F^{-1}\left\{\frac{\frac{2\lambda }{\beta }F\left(t_{i}\right)+F^{*}\left(1\right)F\left(W\right)}{\frac{2\lambda }{\beta }+F^{*}\left(1\right)F\left(1\right)}\right\}$$

where F(1) is the FFT of the dirac delta function.

This process is repeated until convergence with a fixed number of iterations that depends on β and λ. In our case, the algorithm converges after ten iterations with β=0.01 and λ=5 and they were chosen based on numerical experiments. For more information on the convergence conditions of this type of functional and its regularization (with a single regularization term), we recommend the work of Wang [18].

The Figure 2 shows a comparison between the transmission obtained from the optimization model and the real depth image estimated by Zhang [19].

## 3. Fusion

**Figure 3:**
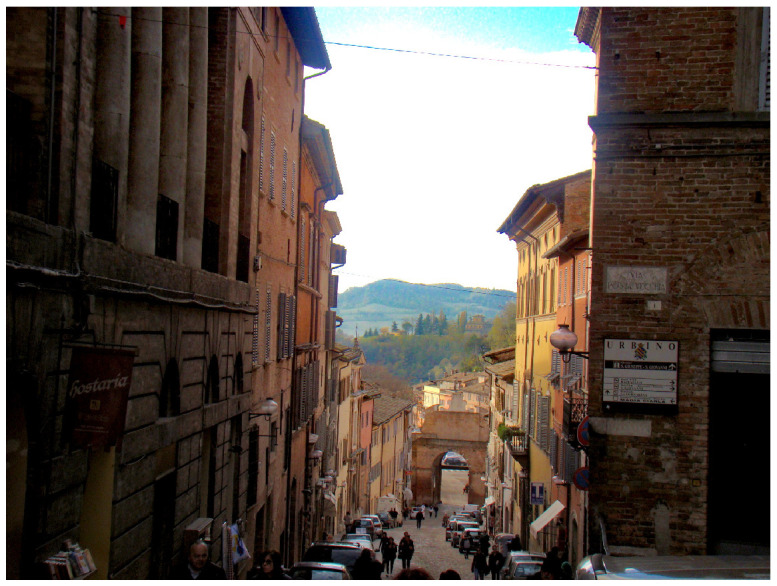
Image where sky restoration failed.

As mentioned earlier, despite the improvement in the estimation of $A_{s}$ proposed in the Section 2.1, the results are insufficient because the color of the haze is not homogeneous throughout the scene. The Figure 3 shows an example of this situation where any dehazing processing using a simple scalar for atmospheric light fails in restoring the sky.

To solve this problem, we introduce the fusion of several values of $A_{s}$ with the multiresolution approach which has the advantage of extracting the relevant information at each level of resolution.

In the literature, this is known as the pyramid fusion, consisting of a set of successive levels of resolution that are increasingly reduced compared to the original image. We propose to use the approach introduced by Burt [20] based on the Gaussian and Laplacian pyramids.

In this section, we explain how the two pyramids are used for fusion using two different methods. First by combining all possible values of $A_{s}$ with a Gaussian weighting to reduce the dominance of images from low-values of $A_{s}$. Secondly, by limiting ourselves to a weightless fusion of some values of the atmospheric lights neighboring the previously estimated value $A_{s}$. This second approach allows us to gain in calculation time and memory needed to store all the images while keeping the same performance.

Note:

We noted the estimated atmospheric light value introduced in Section 2 as $A_{s}$ for $A_{scalar}$ and in the rest of the article, *A* will be considered as a vector of values between 0 and 255.

### 3.1. Gaussian Pyramid

The Gaussian pyramid *G* is applied to the weight map with a resolution of *k* and a depth equal to *N* the number of images to fuse. Considering a standard weight map *W*, we have:

(18) $$W_{i,j}^{n}:=\frac{W_{i,j}^{n}}{\sum_{n=1}^{N}W_{i,j}^{n}}$$

where $i=[0,2^{k}]$ is the number of columns, $j=[0,2^{k}]$ is the number of rows and n=[1,N] is the number of images to fuse.

The multiresolution representation of this weight map can be written as:

(19) $$\begin{array}{c} \begin{array}{cc} G_{l}\left(i,j\right)\left\{W\right\} & =\Delta (G_{l+1}\left(i,j\right))\left\{W\right\}\\ \; & =\sum_{m=-2}^{2}\sum_{n=-2}^{2}w_{g}\;.\;G_{l+1}\left(2i+m,2j+n\right)\left\{W\right\} \end{array} \end{array}$$

where $G_{l}$ represents the level *l* of the image in the pyramid, $w_{g}$ is the Gaussian kernel coefficients, l=[0,lev−1] where lev is the level of the pyramid defined by: $lev=\frac{log(min(cols,rows))}{log(2)}=\frac{log(2^{k})}{log(2)}$ and $i=0,\ldots ,2^{k}-1$, $j=0,\ldots ,2^{k}-1$.

### 3.2. Laplacian Pyramid

The Laplacian pyramid *L* is simply defined as the Gaussian pyramid *G* except that each level corresponds to the pixel to pixel difference at two levels of the Gaussian pyramid. This pyramid is then applied to the images of the radiance J(x) to be fused:

(20) $$L_{l}\left\{J\right\}=G_{l}-\nabla \left(G_{l-1}\right)\left\{J\right\}$$

where ∇ is the expansion operator from level l−1 to the level *l*.

### 3.3. Fusion Process

After the transmission estimation and the initialization of the atmospheric light $A_{s}$, we present in this section our pyramidal fusion to merge images from multiple *A* values. We propose the total fusion of 256 images for all A=[0,255] and then its optimized version of fusion using only 2δ+1 images for A=[A−δ,A+δ]. The radiances J(x) based on 256 values is generated using the Koschmieder diffusion Equation (2), such as:

(21) $$J_{i}\left(x\right)=\frac{I\left(x\right)-A_{i}}{t(x)}+A_{i},\;i=\left[0,\;255\right]$$

#### 3.3.1. Pyramidal Fusion

**Figure 4:**
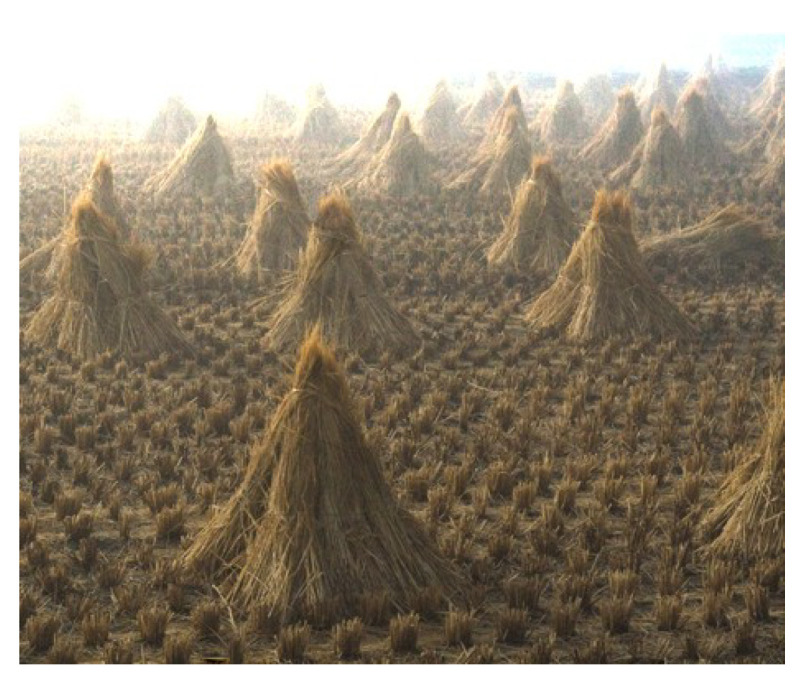
The R(x) image obtained with a fusion of 255 images and $W={\left[1\right]}^{n}$ unit weights.

The fusion consists of multiplying the two pyramids, the Gaussian pyramid *G* of the weight map and the Laplacian pyramid *L* of the radiances (see Figure 4):

(22) $$R\left(x\right)=\sum_{l=1}^{lev}\sum_{n=1}^{N}G{\left\{W\right\}}_{n}^{l}L{\left\{J\right\}}_{n}^{l}$$

In most situations, the previously estimated atmospheric light has values converging to 255, so its value is never centered in the range [0,255]. Thereby, the fused radiance R(x) is dominated by the structure of the images which corresponds to values less than $A_{s}$. This explains the "burned" area on the top of the Figure 4. To overcome this problem, we propose to introduce Gaussian weights *W* such as:

$$W∼N(A_{s},\;255-A_{s})\;$$

**Figure 5:**
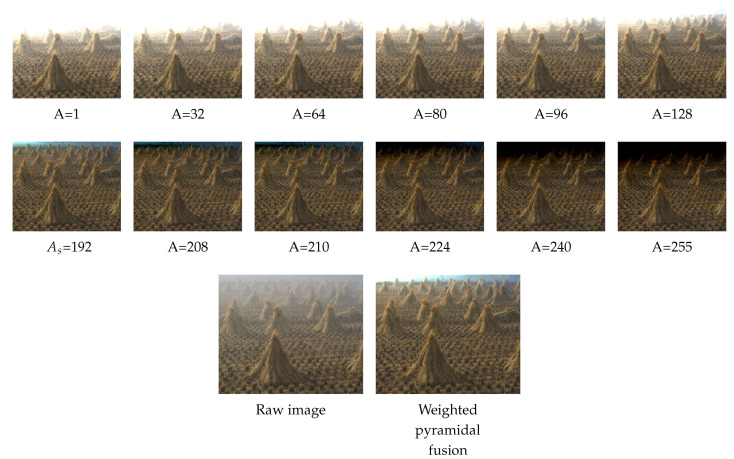
Sample images of the radiances $J_{i}\left(x\right)$ and their fusion image R(x). The atmospheric light estimated is $A_{s}=192$. So, the image with $A_{s}=192$ is our dehazing approach without fusion.

After the application of the Gaussian weights W∼N(193,52), we recover the burned area of the Figure 4 (see Figure 5).

#### 3.3.2. Reduced Pyramidal Fusion

The fusion proposed previously can be expensive in memory because it requires the storage of 256 high-resolution images in memory with their Laplacian levels ($256\times \frac{log(2k)}{log(2)}$ images). In order to reduce the calculation time and the memory needed by more than 10 times, we propose a fusion without weight map *W* and with a reduced set of *A* values.

As previously stated, *A* will take values centered on the estimated value as of Section 2 such as $A=[A_{s}-\delta ,\;A_{s}+\delta ]$, and the weight map will be set to unit values of $W={\left[1\right]}^{n}$.

**Figure 6:**
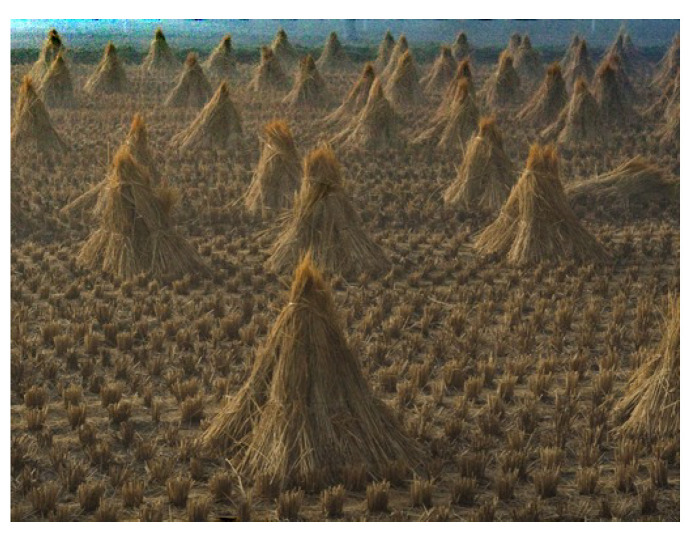
Weightless fusion of 21 radiances. $A=\left[A_{s}-\delta ,\;A_{s}+\delta \right]=\left[193-10,\;193+10\right]=\left[183,\;203\right]$.

The Figure 6 shows, for our test image, the result of the weightless fusion of twenty one radiances $J_{i}\left(x\right)$ of $A_{i}$:

## 4. Results

**Figure 7:**
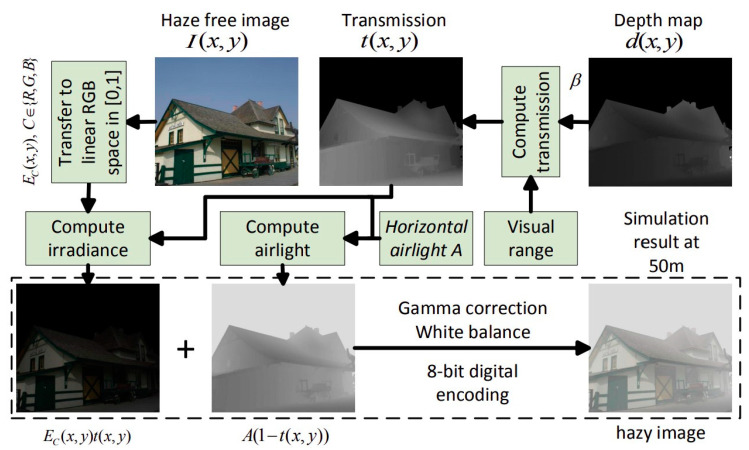
Description of Zhang [19] method for generating the database used in the Figure 8.

Our algorithm achieves good results both quantitatively, according to *SSIM* and *ZNCC* criteria and qualitatively based on the visual quality of the images dehazed. By definition, *SSIM* [21] measures the structural similarity while *ZNCC* [22] computes the centered standardized cross-correlation. These two criteria are applied to images before generating haze and after applying the proposed dehazing method to determine how identical the images are. For this, we use the database of artificially hazy images generated by Zhang [19] based on the visual aspect of the scene proposed by Mccartney [23] (see Figure 7).

The following subsections show the results of our approach comparatively to the state of the art [5,10,15,17,24] on quantitative measures and visually.

### 4.1. Quantitative Results

**Figure 8:**
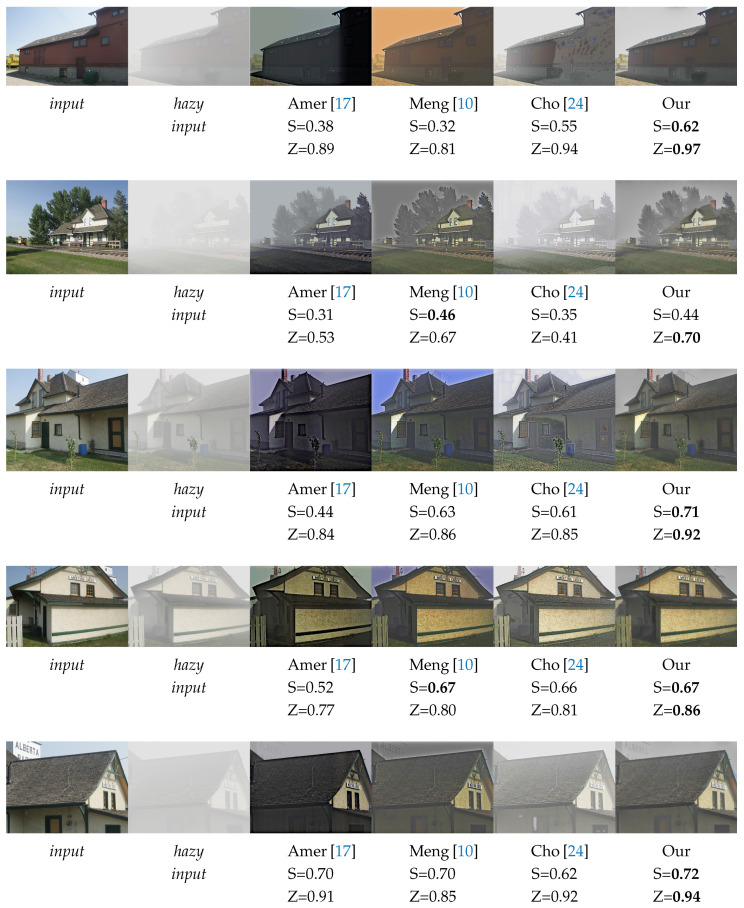
Comparison between five images with their *SSIM (S)* and *ZNCC (Z)* scores. In **bold** the best results for each criterion.

In this section, we present the quantitative results on five different images in comparison to the methods proposed by Meng [10], Amer [17] and Cho [24]. We consider the method to be efficient and likely to have a good visual rendering if it achieves a better score on both *SSIM* and *ZNCC*, usually greater than 0.65 for each criterion. The Figure 8 shows the different scores obtained on five images taken from Zhang’s database.

The Figure 8 shows that the proposed approach outperforms the state of the art for the *SSIM* and *ZNCC* measures. Only one image has a lower *SSIM* but very close to the best one. The method of Amer [17] works well when the haze is dense but, on the other hand, it tends to have overall dark images and is totally burned in some areas of the image where the haze is relatively low. This may be due to the Gaussian filter applied to the transmission during its formation; the transmission is then homogeneous over a large region of the image where the haze is not. Cho [24] has a contrary effect on some regions with few hazes and often gets a blue and reddish hue. This is probably due to the method of estimating parameters based on atmospheric light. Meng [10] obtains good *SSIM* and *ZNCC* scores but the weak point is the instability of areas like the sky due to the initialization of the transmission with the value of *A* that is not robust.

We have observed that for high scores of *SSIM* and *ZNCC* simultaneously (greater than 0.65), the images have a rendering very close to a human prediction and the obtained results are convincing. The Figure 8 shows that a single score can easily reach a value close to 1 but the image remains visually degraded. As an example, the first processed image of Cho [24] where the *ZNCC* score is very good (ZNCC=0.94) but still the image presents artifacts on a large area (SSIM=0.55).

Finally, even if the results overcome the state of the art, these measures remain insufficient in front of the visual aspect. For this purpose, we propose in the following subsections a visual comparison between our results and some results from the literature [5,10,15,17].

### 4.2. Qualitative Results

**Figure 9:**
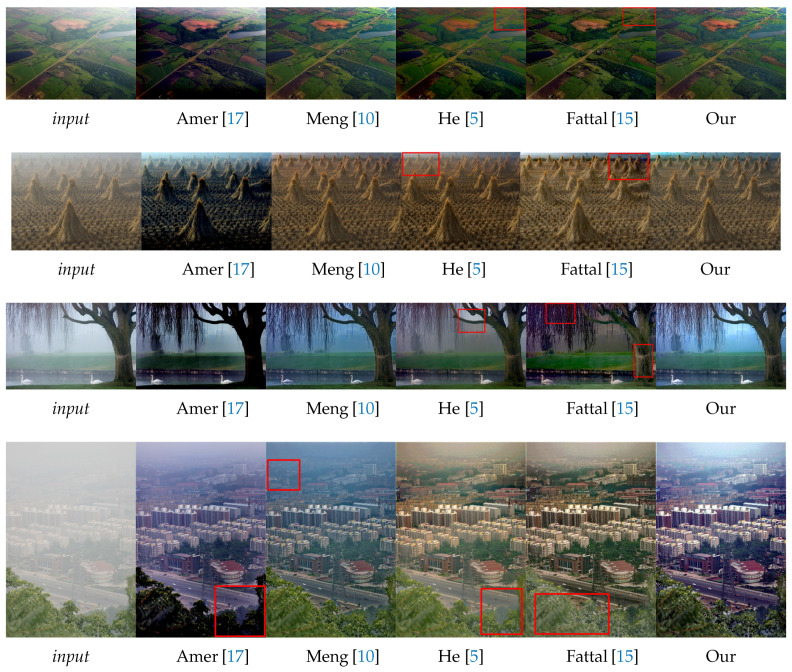
Qualitative results on well known images of the dehazing literature. Note that these images have a large variation of depth. Red boxes indicate dehazing artifacts.

**Figure 10:**
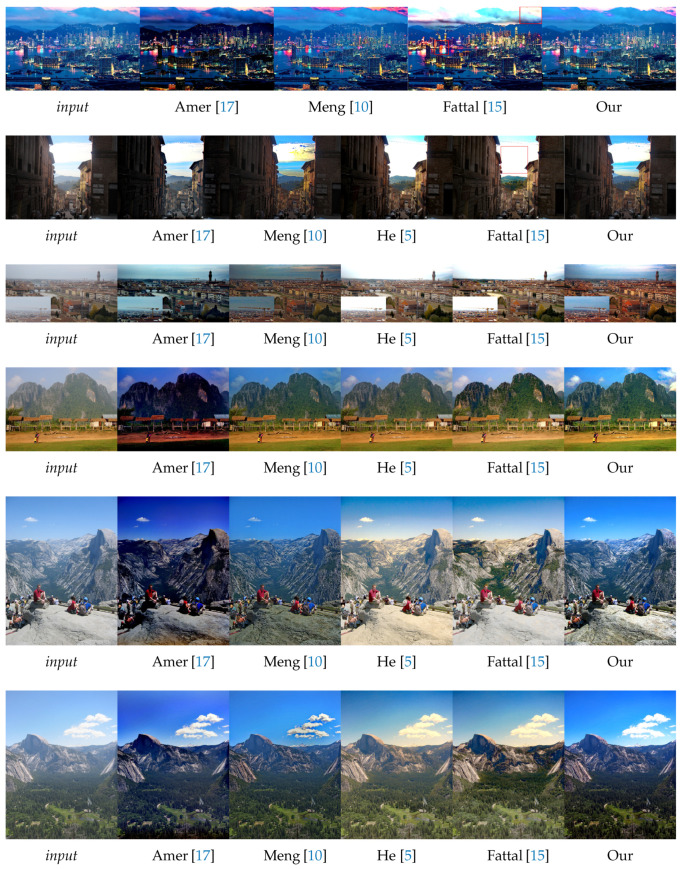
Qualitative results on images with both sky and clouds. Red boxes indicate dehazing artifacts.

**Figure 11:**
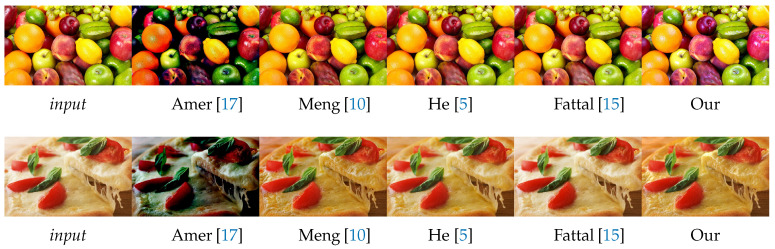
Qualitative results on haze-free images. Red boxes indicate dehazing artifacts.

In this section, we evaluate the visual aspect of our method in comparison with some approaches from the literature considered as a reference in image dehazing [5,15]. So, the comparison between these methods is based on the details and some particular areas of the image. For this reason, we divide the images into three categories: 1. images of landscapes (Figure 9). 2. images with large sky (Figure 10). 3. haze-free images (Figure 11). Those images were chosen based on the fact that it is hard to use one algorithm to restore details from haze regions, successfully process the sky and at the same time not degrade images with no haze.

#### 4.2.1. Landscape Images

The Figure 9 shows that our method produces good visual rendering and bright colors in the most difficult real conditions and in a variety of scenes in terms of contrast, depth and haze density. Thanks to our Gaussian and Laplacian fusion, they give a stable behavior without artifacts.

Fattal [15] significantly improves the result of most situations and, in any case, succeeds in removing the haze from the images. However, the main problem of that approach is the radiance of the images becoming less realistic. We can observe this on the first image where the sky light was removed with the haze or on the third image where the tree is oversaturated, especially the branches.

The method of He [5] is known for its robustness, but it also presents a weak point in the transmission estimation based on the dark channel, as on the second image where the haze in the upper part is underestimated. We can think that the dark channel is badly estimated in regions where no channel in the image converges to a minimum value.

Amer [17] inherits the same positive and negative points of He [5] because their method is based on the dark channel as well but estimated differently with a Gaussian filter. This one is known for its smoothing effect which can cause a false estimation of transmission that leads to dark images.

#### 4.2.2. Sky Images

The most difficult images to dehaze are usually those with strong contrast differences, images taken at night or hazy-free images. In Figure 10, we demonstrate the effectiveness of our method to improve the quality of the results in these situations, especially those where the sky and clouds are both parts of the scene.

The remarks of the Figure 9 remain correct for this kind of images but some details must be given for sky processing. The methods presented have got fairly similar results for the treatment of the sky except Meng [10] who succeeded in having a good extraction of the sky despite that it is a bit dark and tinted. Amer [17] gets good results on the sky part as well but it is a bit oversaturated as it has been stated before. Even if the state of the art methods perform well, our approach is more stable throughout the images. Indeed, the sky has a different atmospheric light than the rest of the scene and our Gaussian and Laplacian fusion leads to a better estimation of it.

#### 4.2.3. Haze-Free Images

On the Figure 11 we show the results of our approach on two haze-free images. This allows us to see the visual impact of the estimation of the transmission that should converge towards 1.

The difference between the images is small except for Amer [17] which tended to overestimate the haze which does not exist (underestimate the transmission) because the images are very bright. Fattal [15] stated in his article a very small error between the transmission obtained and the desired transmission (t(x)=1) compared to He [5] and this is visually validated in the Figure 11. Meng [10] looks similar to He [5] or to our method, though it is a bit more contrasted on the image of the pizza.

The difficulty of processing haze-free images is due to an overprocessing of the high color pixels by overestimating the haze. In our approach, this ambiguity is bypassed using the Gaussian and Laplacian fusion of multiple values of the atmospheric light.

### 4.3. Computation Time

The computation time was evaluated on color images over an average of 100 iterations to refine the evaluation. The Table 2 summarizes the results for three image resolutions. The program was developed in Matlab 2019a on a 2.80 GHz processor with four cores. Overall, the distribution of the computation time is: 50% for the transmission optimization costs, 40% for the fusion and 10% for the rest of the algorithm ($A_{s}$ estimation, image reconstruction after fusion, etc.).

The Table 3 shows a comparison between the computation time of our method against the state of the art. Fattal [15] reported 5.4 s for processing 1 Megapixel image size with 5 s for maximization of the Gauss–Markov model (GMRF). Amer [17] saves a lot of time because of the one-pass iteration on the frequency domain to perform Gaussian filtering. He [5] presents the longest time spent mostly in soft-matting for the refinement of the transmission.

Even though the values of the computation time depend on many factors such as code language, parallel execution or even GPU implementation, the Table 3 shows that our method stays competitive among the state of the art. Fattal [15] is the second fastest method but still failed when it comes to sky dehazing, see Figure 10. Excluding Amer [17] which does not perform well visually, our method is one of the fastest in terms of the running time while having the best performance in terms of the processing effect.

## 5. Conclusions and Perspective

In this article, we have proposed a new method to deal with dehazing in the most difficult conditions, namely the sky or scenes with a strong variation of contrast. The difficulty comes from the fact that the color of the haze is very similar or identical to the color of the sky which generates a false estimation of the transmission t(x) and thus an image overcontrasted.

To address this problem, first, we have developed a more efficient and faster estimation of atmospheric light $A_{s}$ based on a resized version of the hazed image, and secondly, the fusion of several radiance images from different values of *A* centered on the previously estimated value $A_{s}$. This allows us to process the image indirectly with different values of *A*. The results presented show that our process obtains the best visual rendering (quantitatively ant qualitatively) of realistic quality in terms of color and contrast. Our method has also been tested on underwater images which is a challenging environment where the visibility is very limited.

The outlook for this work will be focused on integrating the values of *A* vector at the beginning of the algorithm, more specifically in the optimization of the transmission. From this, we can fuse images of transmission and atmospheric light. We also propose to integrate a weighted fusion, based on contrast and other criteria, of the radiance images. This will then allow us to generate a weight map that we can reuse for refining the transmission as it could be considered as a second output of the algorithm or even reintegrate it into the algorithm.

## Figures and Tables

**Table 1:** Comparison of running time for the estimation of $A_{s}$. In **bold** the best results.

Resolution	Proposed Method	Meng [10]	He [5], Amer [17]
500×332×3	**0.005205**	0.036404	0.02140
2516×3873×3	**0.208691**	8.541274	3.8477

**Table 2:** Computation time of our algorithm for three resolutions.

Resolution (RGB)	Computation Time (s)
400×600×3	1.5746
600×960×3	3.7901
768×1024×3	5.1347

**Table 3:** Comparison of the computation time of our method against some methods of the literature on a 2.80 GHz machine and 620×460 resolution. (*) It was estimated relative to the reported running time of 5.4 s for 1 Megapixel by Fattal [15].

Methods	Computation Time (s)
Amer [17]	0.28
Fattal [15]	1.54 *
Our	1.96
Meng [10]	4.25
Cho [24]	8.20
He [5]	18.06
